# SG-APSIC1043: Creating an urgent operating room alert using Kaizen automation

**DOI:** 10.1017/ash.2023.113

**Published:** 2023-03-16

**Authors:** Nanthipha Sirijindadirat

**Affiliations:** President, Central Sterilizing Services Association, Bankok, Thailand

## Abstract

**Objectives:** We sought to reduce waiting time for instruments in the operating room, to develop technology for communication between zones, to record data in real time for planning instrument management, and to increase trust and satisfaction of customers. **Methods:** The central sterile supply department (CSSD) provides sterilization of instruments and medical devices, mostly to the following departments: operating room, dental unit, outpatient otolaryngology, and obstetrics and gynecology. The CSSD processes 557,588 units per month, among which 30.05% are for the operating room. In the normal process when the operating room send instruments for decontamination, the operating room staff places stickers on the item or uses a form to document the request. The normal turnaround time was 3 hours and 9 minutes and urgent turnaround time was 2 hours and 14 minutes. For color control, we used white color for normal requests and pink for urgent requests. We redesigned our record keeping using Google forms and sent data on dashboards with real-time alerts in the CSSD application to notify staff of an urgent request. **Results:** Staff in the dirty zone placed an urgent tag on the instrument baskets of the auto-washer. After the door was opened in the clean zone, staff noted the urgent tag and marked a validation sheet, then placed a red unilock on the rigid constrainer. Peel pouches were marked “urgent,” advising sterile storage zone staff to separate the basket from normal baskets and to apply the “urgent” paper form and labelling. **Conclusions:** Turnaround time improved by 15 minutes for urgent instruments. Lead time to export data for analysis increased from 80% to 95.49%. Because of facility design, it was difficult to ensure communication between separate dirty, clean, and sterile storage zones. We redesigned our process using new technology to improve turnaround times, reduce waste, improve communication, and increase productivity.

